# Self‐assembled verteporfin nanoparticles decrease drug efflux by P‐glycoprotein without light activation in drug‐resistant cancer cells

**DOI:** 10.1002/btm2.70136

**Published:** 2026-03-20

**Authors:** Idrisa Rahman, Anju Meda, Kaitlyn A. Moore, Andaleeb Sajid, Suresh V. Ambudkar, Huang Chiao Huang

**Affiliations:** ^1^ Fischell Department of Bioengineering University of Maryland College Park Maryland USA; ^2^ Laboratory of Cell Biology, Center for Cancer Research National Cancer Institute, National Institutes of Health Bethesda Maryland USA

**Keywords:** ABC transporter, cellular ATP levels, drug delivery, light‐independent, mitochondrial metabolism, multidrug resistance, P‐glycoprotein, verteporfin

## Abstract

P‐glycoprotein (P‐gp)‐mediated multidrug resistance (MDR) remains one of the major obstacles to successful chemotherapy for cancer. Previous studies have shown that photodynamic therapy and priming using verteporfin (VP) decrease the drug efflux by P‐gp and improve chemotherapy efficacy. Despite these promising results, the inhibitory effects on P‐gp are reversible, as continuous protein synthesis restores P‐gp expression. The effects of VP under non‐illuminated conditions remain underexplored in the context of P‐gp‐mediated MDR. In this study, we investigate the capacity of non‐illuminated self‐assembled nanoaggregates of VP (NanoVP) to modulate P‐gp efflux function by targeting mitochondrial metabolism in vitro. We found that high concentrations of NanoVP, when exposed to drug‐resistant cells for longer time periods, decrease oxygen consumption rate and adenosine triphosphate (ATP production in mitochondria. We determined a concentration (5 μM) and time point (72 h) for NanoVP treatment that significantly decreases mitochondrial ATP levels with minimal cytotoxicity. This metabolic perturbation results in enhanced accumulation of P‐gp substrates and decreased binding of P‐gp‐specific antibodies, indicating the sustained inhibition of P‐gp function. Notably, NanoVP‐mediated inhibition of P‐gp without light activation enhances the efficacy of chemotherapeutic agents that are P‐gp substrates in drug‐resistant cells. Thus, our findings introduce a novel, light‐independent application for VP as a metabolic priming agent to overcome P‐gp‐mediated MDR and improve chemotherapeutic efficacy in drug‐resistant cancers.

## Graphical abstract

*Sustained P‐glycoprotein (P‐gp) inhibition in drug‐resistant cells*: Schematic representing the treatment conditions to achieve P‐gp inhibition in drug‐resistant cancer cells. In untreated drug‐resistant cells, P‐gp actively effluxes chemotherapeutics outside of cells, causing them to be ineffective. Treatment conditions such as treatment time, verteporfin formulation, and concentration cause transient or sustained P‐gp inhibition indirectly via ATP depletion or directly at the protein level. This image was created using BioRender.com.

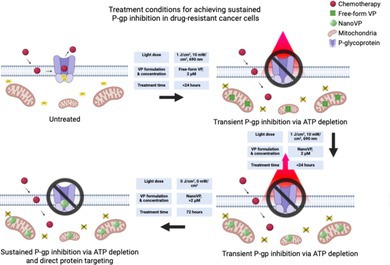

**Translational Impact Statements**

This work demonstrates a novel, light‐independent use of verteporfin (VP) for P‐glycoprotein (P‐gp) inhibition by exploiting the acquired metabolic vulnerabilities in drug‐resistant cells due to P‐gp overexpression. By repurposing the FDA‐approved photosensitizer, VP, we can modulate mitochondrial ATP production without light activation and directly target P‐gp function and enhance the efficacy of chemotherapy in drug‐resistant cells. These findings offer a clinically relevant strategy for sensitizing drug‐resistant tumors to chemotherapy and laying the foundation for next‐generation metabolic co‐therapies in oncology treatment.

## 1 INTRODUCTION

Resistance to chemotherapy is one of the largest challenges to successful therapeutic outcomes clinically.^1^ To make matters worse, chemoresistance can be non‐specific to any one drug or several drugs together, a phenomenon called multidrug resistance (MDR).^1^ One of the most significant drivers of MDR is the overexpression of P‐glycoprotein (P‐gp) on cancer cell surfaces, whereby chemotherapy drugs are actively effluxed out of the cancer cells, preventing them from exhibiting their therapeutic potential.^2^, ^3^, ^4^, ^5^ Traditional methods for overcoming P‐gp‐mediated MDR, such as inhibition by competitive small molecule inhibitors, have had limited clinical success due to unreasonably high dosing requirements and off‐target toxicity.^6^ As such, research into alternative approaches to target P‐gp efflux function has been underway. Photodynamic therapy (PDT) has emerged as a promising approach for overcoming MDR.^7^ PDT reactions are photochemistry‐based, where a photosensitizer is illuminated at a specific wavelength of light, leading to the generation of reactive oxygen species (ROS), which causes cell death.^8^ We have previously shown that photoactivation of the photosensitizer, verteporfin (VP), can target P‐gp directly by causing crosslinking of the proteins at the plasma membrane level.^9^ We also found that photoactivation of VP causes mitochondrial damage that causes ATP depletion in drug‐resistant cells and decreases the efflux function of P‐gp without damaging the protein at the plasma membrane level.^10^, ^11^

In recent years, the use of VP has been investigated as a treatment without photoactivation, largely in the context of glioblastoma, given its invasive nature and likelihood of recurrence, where results have shown significant decreases in growth and viability of cancer cells.^12^, ^13^, ^14^, ^15^, ^16^ Light‐independent VP is also being investigated in a current clinical trial (phase I/II) to treat recurrent, high‐grade, epidermal growth factor receptor (EGFR‐mutated glioblastoma (NCT04590664), using a liposomal formulation of VP.^17^ The proposed mechanism of action for light‐independent VP treatment is the disruption of the Yes‐associated protein (YAP)‐transcriptional enhanced associate domain (TEAD) complex that plays a key role in cancer cell survival and proliferation.^12^ Quinlan et al. found that this mechanism of action is conserved when using NanoVP, a self‐assembled nanoaggregate of VP, compared to a liposomal formulation of VP in a glioblastoma model.^18^, ^19^

Beyond its canonical role in regulating cell proliferation and survival, accumulating evidence indicates that Hippo pathway signaling, through YAP and transcriptional coactivator with PDZ binding motif (TAZ)‐dependent regulation of metabolic gene expression, contributes to the control of mitochondrial function, oxidative phosphorylation (OXPHOS), and ATP production.^20^ In parallel, emerging evidence suggests that VP can also exert light‐independent effects on mitochondrial function that are not fully explained by Hippo modulation. Kuramoto et al. reported that VP treatment induced mitochondrial dysfunction and OXPHOS inhibition by significantly decreasing ATP production at steady state and depolarizing the mitochondrial membrane potential (ΔΨm) in glioma stem cells.^21^ Notably, these results were observed even in the context of YAP knockdown, although YAP knockdown alone did not recapitulate these metabolic effects. These findings raise the possibility that VP‐induced metabolic perturbations may occur through mechanisms that extend beyond canonical YAP‐TEAD signaling. However, the relationship between these two pathways is not completely understood.

These observations were particularly compelling given our previous observations that VP‐mediated photodynamic priming (VP‐PDP) induces mitochondrial dysfunction and ATP depletion in drug‐resistant cells.^10^, ^11^ While VP‐PDP has shown promising but transient inhibition of P‐gp function, a major challenge remains in sustaining this inhibition to maximize therapeutic efficacy. Given the emerging role of VP as a modulator of cellular metabolism, we sought to investigate whether non‐photoactivated VP could provide a complementary, light‐independent strategy to suppress P‐gp function by exploiting the heightened ATP dependence of P‐gp overexpressing drug‐resistant cells to functionally impair efflux activity. Although glioma stem cells exhibit resistance‐like phenotypes, the effects of light‐independent VP on bona fide P‐gp‐overexpressing MDR cancer cells have not been systemically examined. In this study, we evaluate the impact of prolonged exposure to NanoVP on mitochondrial metabolism and P‐gp efflux function in established drug‐resistant breast and ovarian cancer models. While YAP‐TEAD signaling has been implicated in VP activity in other systems, the present work focuses on metabolic and transporter‐level consequences of light‐independent NanoVP exposure, without directly interrogating Hippo pathway signaling.

Furthermore, our previous results show that VP is a substrate for P‐gp^10^ and it is known that substrates can modulate P‐gp activity in a concentration‐dependent manner leading to either inhibition or stimulation of efflux.^9^, ^22^, ^23^ Prior studies have primarily examined VP effects over short exposure times (≤24 h) in the context of YAP signaling and metabolic perturbation.^12^, ^21^ Here, we extend this framework by evaluating higher NanoVP concentrations and prolonged exposure to determine whether sustained metabolic stress can inhibit P‐gp function. Our goal is to inhibit P‐gp both directly, through prolonged interaction with the substrate‐binding pocket, and indirectly, by exploiting collateral metabolic vulnerabilities associated with P‐gp overexpression. Collectively, this work addresses a critical knowledge gap by exploring NanoVP as a light‐independent, metabolic priming agent to modulate MDR, expanding the therapeutic scope of VP beyond its traditional role as a photosensitizer.

## 2 RESULTS AND DISCUSSION

### 2.1 NanoVP decreases oxygen consumption rate, extracellular acidification rate, and mitochondrial‐ and glycolytic‐ATP production rates without light activation

**FIGURE 1:**
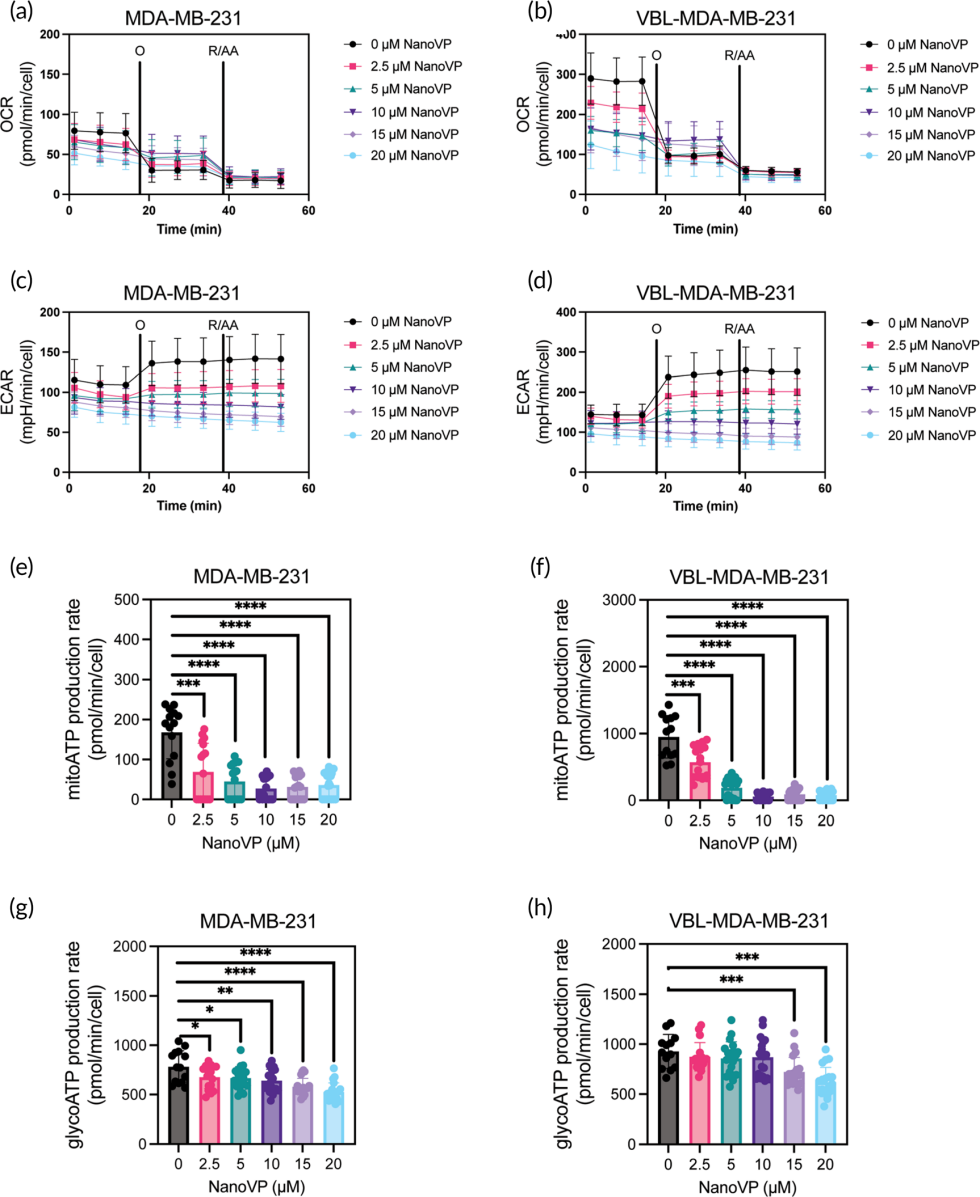
Nanoaggregates of verteporfin (NanoVP) decreases oxygen consumption rate (OCR), extracellular acidification rate (ECAR), mitochondrial ATP (mitoATP) and glycolytic‐ATP (glycoATP) production rates in chemosensitive and chemoresistant cells without light activation. Cells were treated with NanoVP (0–20 μM) for 24 h prior to the assay. The Agilent Seahorse RealTime ATP assay was used to measure OCR in chemosensitive (a) and chemoresistant cells (b). ECAR was also measured from this assay in chemosensitive (c) and chemoresistant cells (d). mitoATP production rates were calculated for chemosensitive (e) and chemoresistant cells (f). The glycoATP production rates were calculated as described in the methods for chemosensitive (g) and chemoresistant cells (h). Data presented as mean ± standard deviation (SD) values (*n* = 3), *p ≤ 0.05, **p ≤ 0.01, ***p ≤ 0.001, ****p ≤ 0.0001, two‐tailed *t*‐test.

To determine the effect of non‐photoactivated NanoVP on cellular energetics, we began by screening concentrations of NanoVP (0–20 μM) following a 24‐h treatment in two triple‐negative breast cancer (TNBC) cell lines. MDA‐MB‐231 cells are the parental, non‐P‐gp‐expressing cell line and VBL‐MDA‐MB‐231 are the vinblastine‐resistant, P‐gp‐overexpressing subline. NanoVP particle size, polydispersity index (PDI), and surface charge were characterized by dynamic light scattering (DLS) and are summarized in Table S1, consistent with prior characterization methods.^18^ We used the Agilent Seahorse Extracellular Flux (XF) Real Time ATP assay to measure changes to oxygen consumption rate (OCR), extracellular acidification rate (ECAR), and ATP production in mitochondria (OXPHOS) and glycolysis. This assay was used as a functional screening tool to assess how mitochondrial and glycolytic‐ATP (glycoATP) production redistribute in response to NanoVP exposure, rather than to quantify basal or maximal glycolytic capacity. The results revealed that as the concentration of NanoVP increased, the baseline level of OCR progressively decreased. We found that the cells lose their ability to respond to the inhibition of the electron transport chain (ETC) by the addition of oligomycin but not with rotenone + antimycin A in both sensitive MDA‐MB‐231 cells (Figure 1a) and resistant VBL‐MDA‐MB‐231 cells (Figure 1b). This indicates an overall suppression of OXPHOS prior to ETC inhibition.

The lack of response in OCR after oligomycin injection suggests that ATP‐linked respiration is already substantially impaired following NanoVP treatment. However, because Seahorse analysis reports functional changes in respiration rather than direct molecular interactions, these data do not allow assignment of a specific ETC complex as a direct NanoVP target. The observed decrease in OCR after rotenone + antimycin A indicates that ETC complexes I and III remain functionally responsive under these conditions, suggesting that NanoVP does not primarily disrupt electron flux at these sites. Kuramoto et al. previously evaluated ETC vulnerabilities in glioma stem cells using complex‐specific inhibitors and reported sensitivity at complexes III and IV.^21^ In contrast, our findings argue against complex III as a dominant site of disruption in these breast cancer models, leaving downstream components of OXPHOS as potential contributors.

One possible explanation for the observed respiratory phenotype is interference with electron transfer downstream of complex III. A study by Eales et al. showed that VP binds to free iron because of its similar structure to iron‐binding porphyrins.^24^ In the ETC, cytochrome C facilitates the transfer of electrons from complex III to complex IV via its covalently linked heme group.^25^ Disruption of this process could impair overall electron flow and indirectly reduce ATP synthesis without directly inhibiting individual ETC complexes.^26^ Importantly, this interpretation remains speculative, and biochemical assays would be required to confirm any direct interaction between VP and specific mitochondrial components.

Consistent with the OCR findings, increasing NanoVP concentrations also reduced baseline ECAR in both MDA‐MB‐231 cells (Figure 1c) and VBL‐MDA‐MB‐231 cells (Figure 1d). As expected, rotenone plus antimycin A, which are used to completely inhibit mitochondrial respiration and assess maximal glycolytic compensation, did not significantly alter ECAR, indicating that NanoVP treatment does not elicit an acute glycolytic upregulation under these conditions. Together, these results support a model in which light‐independent NanoVP exposure suppresses mitochondrial respiration without inducing a glycolytic response that would offset the loss of oxidative phosphorylation.

We next quantified ATP production rates from mitochondrial respiration and glycolysis. Mitochondrial ATP production rates (mitoATP) significantly decreased in MDA‐MB‐231 cells where we observed a 58.9% decrease (Figure 1e) and a 40.1% decrease in VBL‐MDA‐MB‐231 cells (Figure 1f) at the lowest tested concentration of NanoVP (2.5 μM). A 78.6% decrease in mitoATP production rates was observed in MDA‐MB‐231 cells at the highest tested concentration of NanoVP (20 μM). A 91.8% decrease in mitoATP production rates was observed in VBL‐MDA‐MB‐231 cells at 20 μM NanoVP. At higher NanoVP concentrations, mitoATP suppression reached a plateau, with no statistically significant differences observed between 10 and 20 μM in MDA‐MB‐231 cells and between 15 and 20 μM in VBL‐MDA‐MB‐231 cells (Figure 1e,f). These results align with previous studies showing that mitochondrial respiration is a primary target of non‐photoactivated VP after 24 h.^12^, ^21^

In contrast, moderate reductions in glycoATP production rates were observed that were dependent on NanoVP concentration. We observed a 13.3% decrease in glycoATP in MDA‐MB‐231 cells at 2.5 μM NanoVP, which was significant compared to the untreated control, and decreased further in a dose‐dependent manner to 32.6% at 20 μM (Figure 1g). These results suggest that glycolytic pathways are affected secondarily or adaptively rather than serving as the primary metabolic target. A study by Chen et al. demonstrated that glycolysis is upregulated in breast cancer cells, and VP treatment for 48 and 72 h inhibited glycolysis.^27^ They attributed this effect largely to YAP inhibition, showing that YAP upregulation correlated with increased glycolysis, and that YAP knockdown significantly reduced ECAR as a measure of glycolytic activity.^27^ In the present study, since we did not directly investigate YAP signaling, the contribution of Hippo pathway modulation to glycolytic changes cannot be excluded.

Notably, glycoATP suppression was more pronounced in VBL‐MDA‐MB‐231 cells at higher NanoVP concentrations, with glycoATP decreasing by 22.1% at 15 μM and further decreasing by 33.1% at 20 μM (Figure 1h). This suggests that while drug‐resistant cells may rely more heavily on glycolytic ATP production to support the increased energetic demands imposed by P‐gp overexpression, NanoVP treatment preferentially suppresses mitochondrial ATP production at lower concentrations, while glycolytic ATP production is comparatively preserved. At higher NanoVP concentrations, glycolytic ATP production also declines, indicating that this compensatory capacity becomes progressively limited. Collectively, these findings further highlight the metabolic plasticity of drug‐resistant cells and suggest that limiting their ability to compensate for mitochondrial dysfunction may represent an effective strategy for impairing P‐gp function in MDR cancers.

### 2.2 NanoVP decreases the viability of drug‐resistant cells

**FIGURE 2:**
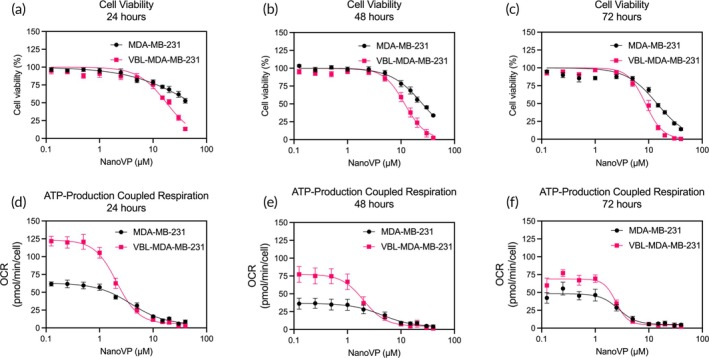
Nanoaggregates of verteporfin (NanoVP) in the absence of light decreases cell viability and ATP‐production‐coupled respiration in chemosensitive and chemoresistant cells from 24 to 72 h in triple‐negative breast cancer cells. Cells were treated with 0–40 μM NanoVP for 24, 48, or 72 h. CellTiter‐Fluor was used to measure cell viability after treatment in MDA‐MB‐231 and VBL‐MDA‐MB‐231 cells after 24 h (a), 48 h (b), and 72 h (c). The Agilent Seahorse MitoStress Test assay kit was used to measure changes in mitochondrial oxygen consumption rate and ATP‐production‐coupled respiration from 24 h (d), 48 h (e), and 72 h (f) in MDA‐MB‐231 and VBL‐MDA‐MB‐231. Data presented as mean ± SD values (*n* = 3), non‐linear regression analysis. OCR, oxygen consumption rate.

We next aimed to determine if the previously observed decreases in OCR, ECAR, and ATP production were a result of a decrease in cell viability. If not, we aimed to identify a concentration of NanoVP at which there was a significant decrease in ATP production levels so that we could measure changes in P‐gp expression and function at that concentration. We also aimed to screen longer time points to determine the optimal time to test the NanoVP concentration that would allow us to evaluate changes in P‐gp expression and function. We tested concentrations of 0–40 μM NanoVP and screened three time points: 24‐, 48‐, and 72‐h. We found that the half maximal inhibitory concentration (IC_50_) values in VBL‐MDA‐MB‐231 cells were lower than the IC_50_ values for MDA‐MB‐231 cells after treatment with NanoVP at all tested time points (Figure 2a–c). The IC_50_ values are summarized in Table 1. The IC_50_ values with all the tested cell lines were at least four times higher than 2.5 μM, where significant reductions in mitoATP were observed, indicating most of the cells were alive at the 2.5 μM condition, so the effects on mitoATP were observed in live cells.

**TABLE 1:** Summarizing the IC_30_ and the IC_50_ values for cell viability and ATP‐production‐coupled respiration in triple‐negative breast cancer cells after a 24‐ to 72‐h incubation with 0–40 μM nanoaggregates of verteporfin without photoactivation.

		IC_30_ (μM)	IC_50_ (μM)	
		Cell Viability	Cell Viability	ATP production
24 h	MDA‐MB‐231	16.9 ± 3.03	54.2 ± 7.84	3.89 ± 0.70
	VBL‐MDA‐MB‐231	10.5 ± 1.60	17.8 ± 0.94	2.08 ± 0.19
48 h	MDA‐MB‐231	14.2 ± 1.48	25.2 ± 1.06	4.47 ± 2.21
	VBL‐MDA‐MB‐231	8.0 ± 1.12	11.7 ± 0.57	1.99 ± 0.39
72 h	MDA‐MB‐231	8.2 ± 1.15	14.3 ± 0.69	2.72 ± 0.50
	VBL‐MDA‐MB‐231	6.3 ± 0.67	8.7 ± 0.34	2.44 ± 0.22

We chose to focus subsequent metabolic analyses on mitoATP production using the Agilent Seahorse XF MitoStress Test Assay because the initial screening revealed a pronounced and dose‐sensitive reduction in mitoATP at low NanoVP concentrations (≥2.5 μM) in both parental and drug‐resistant cells, whereas reductions in glycoATP production in drug‐resistant cells were only observed at substantially higher NanoVP concentrations (≥15 μM) (Figure 1). Based on this differential sensitivity in the drug‐resistant models relevant to P‐gp function, mitochondrial metabolism was prioritized as the primary metabolic process affected by NanoVP under the conditions used for downstream P‐gp functional studies.

The MitoStress test assay revealed that the baseline level of ATP‐production‐coupled respiration is always higher in the drug‐resistant cells due to the increased energy demand in the drug‐resistant cells because of P‐gp overexpression. The ATP‐production‐coupled respiration levels decrease over time after 24‐ (Figure 2d), 48‐ (Figure 2e) and 72‐h (Figure 2f) incubations with up to 40 μM NanoVP. The IC_50_ values for ATP‐production‐coupled respiration are also summarized in Table 1. Interestingly, we observed decreases in baseline ATP‐production‐coupled respiration levels in the drug‐resistant cells starting at 48 h and continuing to decrease at 72 h. While the underlying cause of this time‐dependent decrease was not directly interrogated, one potential contributing factor may be progressive nutrient limitation under extended culture conditions, particularly given the elevated energetic demands associated with P‐gp overexpression. Consistent with this possibility, prior studies have shown that prolonged culture can lead to glucose depletion and altered mitochondrial respiration in cancer cells.^28^ Importantly, regardless of this time‐dependent baseline shift, increasing concentrations of NanoVP consistently induced an additional, dose‐dependent reduction in ATP‐production‐coupled respiration, supporting a direct metabolic impact of NanoVP under the conditions tested.

We therefore chose to use the 30% inhibitory concentration (IC_30_) values of NanoVP after 72 h to investigate the effects on P‐gp expression and function at those concentrations because this time point represents a state where the cells are the most critically ATP‐depleted, with limited cytotoxicity. In contrast, IC_50_ values are used to benchmark cytotoxic responses and compare ATP‐production‐coupled respiration across cell lines. The corresponding IC_30_ and IC_50_ values are summarized in Table 1.

**FIGURE 3:**
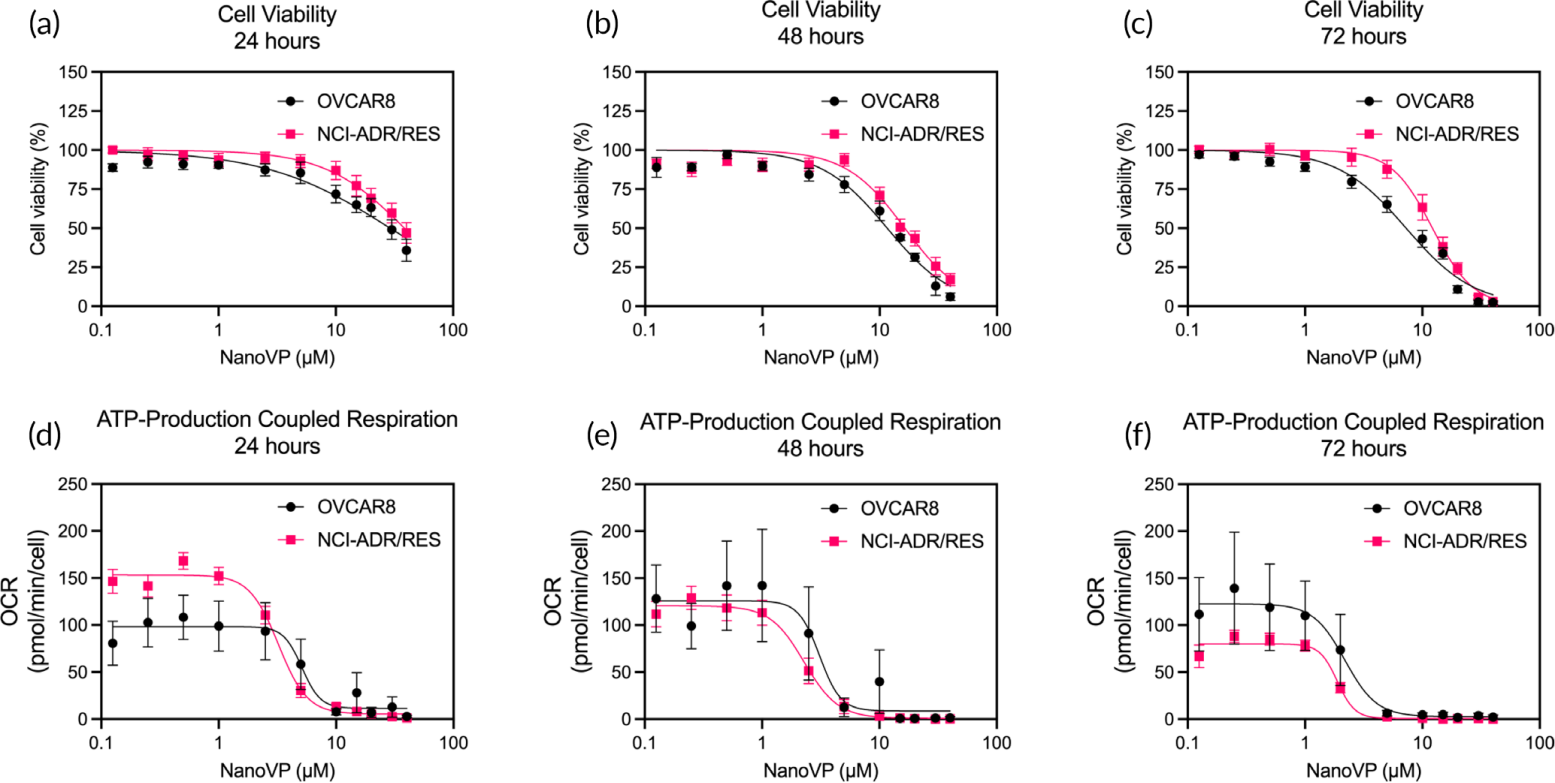
Nanoaggregates of verteporfin (NanoVP) decreases cell viability and ATP‐production‐coupled respiration in chemosensitive and chemoresistant ovarian cancer cells from 24 to 72 h. Cells were treated with 0–40 μM NanoVP for 24, 48 or 72 h. CellTiter‐Fluor was used to measure cell viability after treatment in OVCAR8 and NCI‐ADR/RES cells after 24 h (a), 48 h (b) and 72 h (c). The Agilent Seahorse MitoStress Test assay kit was used to measure changes in mitochondrial oxygen consumption rate and ATP‐production‐coupled respiration from 24 h (d), 48 h (e), and 72 h (f) in MDA‐MB‐231 and VBL‐MDA‐MB‐231. Data presented as mean ± SD values (*n* = 3), non‐linear regression analysis. OCR, oxygen consumption rate.

We also measured the changes in cell viability and ATP production‐coupled respiration in an ovarian cancer cell pair with OVCAR8 as the parental, non‐P‐gp‐expressing cell line and NCI‐ADR/RES as the doxorubicin‐resistant, P‐gp overexpressing subline. Interestingly, the parental cell line was observed to be more sensitive to the NanoVP treatment at all three time points: 24 h (Figure 3a), 48 h (Figure 3b), and 72 h (Figure 3c). The IC_50_ values are summarized in Table 2. We found that the ATP‐production‐coupled respiration levels were higher in the drug‐resistant cells compared to parental cells at 24 h (Figure 3d), were the same between parental and drug‐resistant cells at 48 h (Figure 3e), and were lower in drug‐resistant cells compared to parental cells at 72 h (Figure 3f). The IC_50_ values for ATP‐production‐coupled respiration are also summarized in Table 2. Similar to the TNBC cell pair, we chose to use the IC_30_ values of NanoVP after 72 h to investigate the effects on P‐gp expression and function in the ovarian cancer cell pair. The NanoVP IC_30_ values obtained with OVCAR8 and NCI‐ADR/RES cells are summarized in Table 2.

**TABLE 2:** Summarizing the IC_30_ values and the IC_50_ values for cell viability and ATP‐production‐coupled respiration in ovarian cancer cells after a 24‐ to 72‐h incubation with 0–40 μM nanoaggregates of verteporfin in the absence of photoactivation.

		IC_30_ (μM)	IC_50_ (μM)	
		Cell Viability	Cell Viability	ATP production
24 h	OVCAR‐8	10.4 ± 2.69	29.1 ± 3.73	5.11 ± 0.99
	NCI‐ADR/RES	19.9 ± 3.44	38.5 ± 4.12	3.23 ± 0.20
48 h	OVCAR‐8	6.7 ± 1.20	11.6 ± 0.71	3.09 ± 1.01
	NCI‐ADR/RES	10.03 ± 1.60	16.4 ± 0.91	2.28 ± 0.27
72 h	OVCAR‐8	4.1 ± 0.63	7.28 ± 0.40	2.19 ± 0.85
	NCI‐ADR/RES	8.7 ± 1.09	12.2 ± 0.53	1.90 ± 0.14

To balance metabolic impact with cell viability, we selected 5 μM NanoVP after 72 h because it was lower than the IC_30_ for both drug‐resistant cell lines, ensuring that enough live cells would remain to determine the effect of non‐photoactivated NanoVP on P‐gp expression and function. After a 72‐h treatment with 5 μM NanoVP, we observed a 69.1% decrease in ATP‐production‐coupled respiration in MDA‐MB‐231 cells and an 83.6% decrease in VBL‐MDA‐MB‐231 cells (Figure 2f). We observed a 94.4% decrease in ATP‐production‐coupled respiration in OVCAR8 cells and a 96.3% decrease in NCI‐ADR/RES cells (Figure 3f). These decreased levels of ATP‐production‐coupled respiration, but high levels of cell viability, also show that NanoVP exposure after 72 h is not toxic to cells, but significantly modulates their metabolic state, making this concentration and time point ideal for analyzing P‐gp function and expression levels.

### 2.3 Intracellular NanoVP retention is not significantly different between TNBC and ovarian cancer cell lines up to 72 h

**FIGURE 4:**
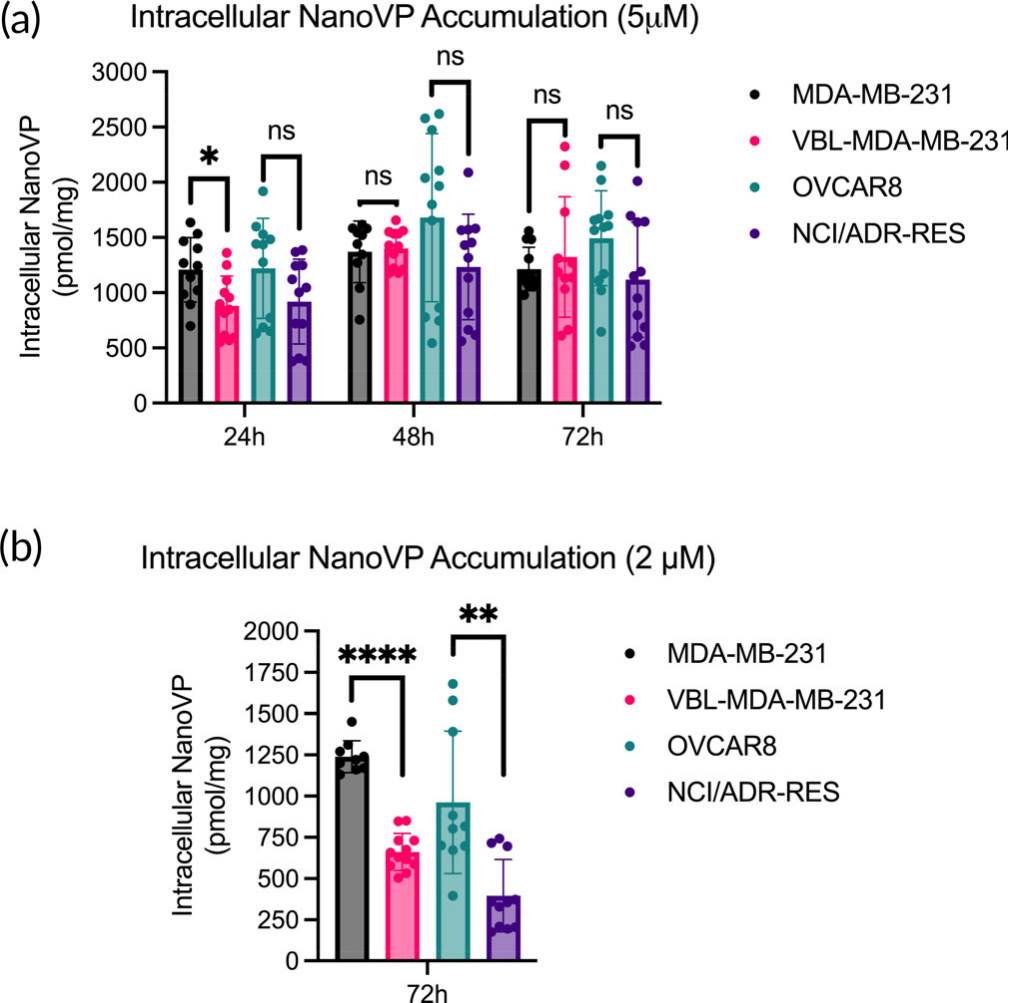
Verteporfin (VP) is not effluxed by P‐glycoprotein in drug‐resistant cells after treatment with 5 μM nanoaggregates of VP (NanoVP) for 24–72 h. Cells were treated with 5 μM NanoVP (a) for 24, 48, and 72 h and 2 μM NanoVP for 72 h (b) and were then lysed, and NanoVP concentration was measured and normalized to total protein content. Data presented as mean ± SD values (*n* = 3), *p ≤ 0.05, **p ≤ 0.01, ****p ≤ 0.0001, two‐tailed *t*‐test.

We observed differences in sensitivity to NanoVP treatment between TNBC and ovarian cancer cells, as reflected by differences in cell viability. We hypothesized that this result was due to differences in NanoVP uptake in different cell lines. We performed a VP extraction assay to determine the levels of intracellular VP accumulation when delivered by NanoVP after 24, 48, and 72 h using 5 μM NanoVP based on the previously selected concentration (Figure 4a). The results revealed that the only statistically significant difference in accumulation was between MDA‐MB‐231 cells and VBL‐MDA‐MB‐231 cells at 24 h. At 24 h, we observed that 72.9% of VP was accumulated in VBL‐MDA‐MB‐231 cells compared to the parental control. We expected that VBL‐MDA‐MB‐231 cells would have a higher level of uptake because of their increased sensitivity shown by the cell viability assay; however, this was not observed. VP uptake in NCI‐ADR/RES cells was lower than in OVCAR8 cells at every time point, though the difference was not statistically significant. Together, these findings indicate that intracellular VP accumulation alone does not predict sensitivity to NanoVP‐induced cytotoxicity. Instead, differential responses across cell lines likely reflect downstream cellular context rather than differences in intracellular drug abundance. Importantly, these accumulation studies were performed to assess whether differential NanoVP uptake could explain cell line‐specific sensitivity, rather than to define the optimal temporal window for NanoVP‐mediated P‐gp inhibition.

One potential explanation is the difference in hormone receptor expression (estrogen, progesterone, and human epidermal growth receptor 2 (HER2)) between the TNBC and ovarian cancer cells. Hormone receptor status has been shown to impact drug sensitivity, as estrogen and progesterone receptor signaling can modulate various survival pathways, including those involved in apoptosis and metabolism.^29^, ^30^, ^31^, ^32^ While TNBC cells lack hormone receptors, they may compensate through alternative survival pathways, which could alter their susceptibility to VP‐induced metabolic disruption.^33^ Additionally, baseline differences in Hippo pathway activity between the cell lines may also contribute to the observed differences in sensitivity, as the Hippo pathway plays a key role in regulating cell proliferation and survival. Given VP's known role in disrupting YAP‐TEAD interactions, differential pathway activity at baseline could influence how each cell line responds to treatment.^34^ However, Hippo signaling was not directly measured in the present study; thus, this hypothesis requires further investigation to determine whether its modulation plays a direct role in VP‐induced cell death in drug‐resistant TNBC cells.

Since we observed a loss of VP efflux from P‐gp at 5 μM following sustained NanoVP exposure with both drug‐resistant cell lines, we wanted to determine whether the observed loss of efflux reflected a concentration‐dependent threshold under sustained exposure. To test this, we performed the VP extraction assay using 2 μM VP for a 72‐h treatment. The 72‐h time point was selected as a stringent exposure condition to evaluate concentration dependence under maximal metabolic stress, rather than to map the temporal onset of efflux inhibition. The results revealed that VP accumulation was significantly lower in drug‐resistant VBL‐MDA‐MB‐231 cells and NCI‐ADR/RES cells compared to MDA‐MB‐231 cells and OVCAR8 cells, respectively, after a 72‐h treatment (Figure 4b). In VBL‐MDA‐MB‐231 cells, 53.4% VP was retained in cells, indicating 46.6% efflux from P‐gp after 72 h. In NCI‐ADR/RES cells, 41.2% VP was retained in cells, indicating 58.8% efflux from P‐gp after 72 h. These findings suggest that P‐gp remains functional at 2 μM NanoVP, as substantial efflux was observed in drug‐resistant cells. However, the loss of P‐gp‐mediated efflux at 5 μM NanoVP suggests a concentration threshold at which sustained exposure leads to inhibition of P‐gp. This comparison highlights a key concentration‐dependent threshold effect. While sustained exposure (72 h) to VP at 2 μM results in significant efflux in drug‐resistant cells, sustained exposure at 5 μM at the same time point leads to P‐gp inhibition in drug‐resistant cells. The disappearance of efflux at 5 μM is consistent with prior reports of biphasic behavior observed for certain P‐gp substrates, such as verapamil, where low concentrations stimulate P‐gp function, while higher concentrations inhibit it.^35^ While additional intermediate concentrations would be required to fully characterize a biphasic response profile, the present data identify a functionally relevant concentration threshold at which sustained NanoVP exposure is associated with loss of P‐gp‐mediated efflux.

### 2.4 NanoVP increases intracellular substrate retention in drug‐resistant cells after 72 h

**FIGURE 5:**
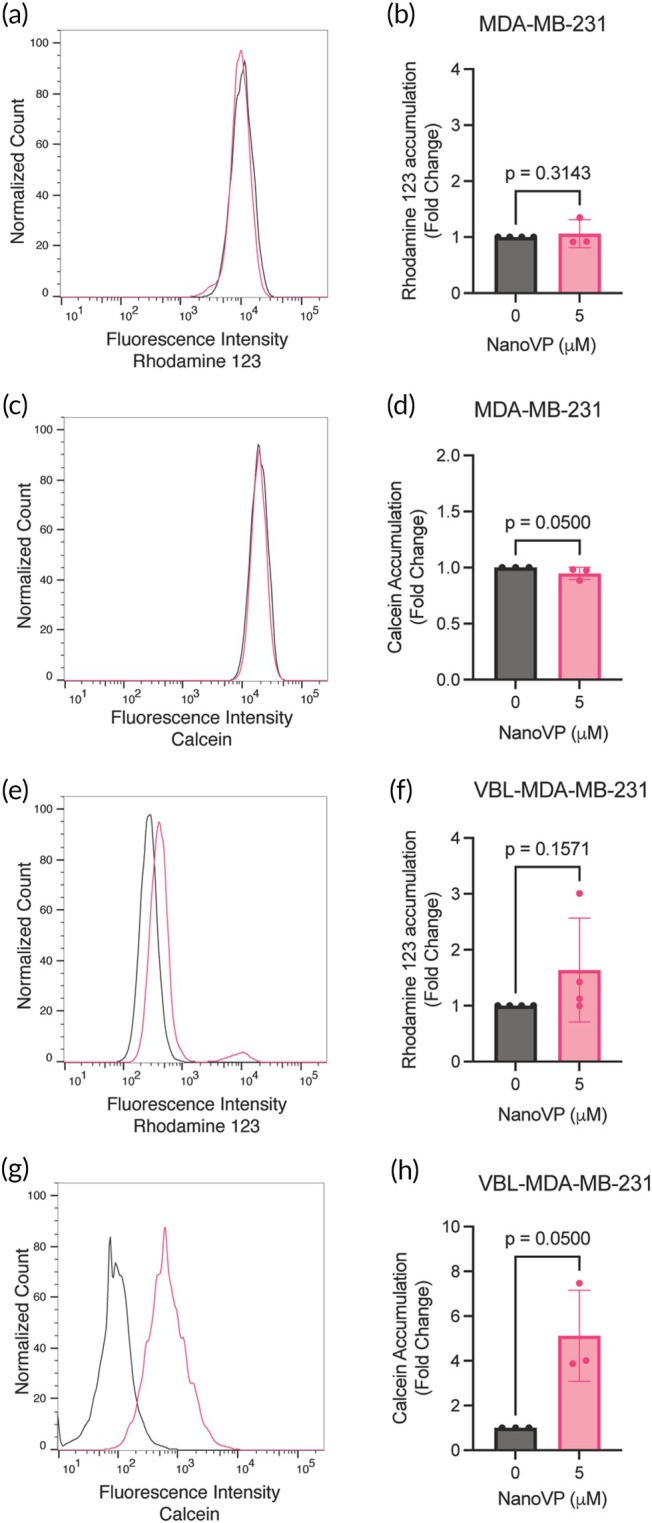
Nanoaggregates of verteporfin (NanoVP) treatment increases the accumulation of P‐glycoprotein substrates after 72 h in drug‐resistant triple‐negative breast cancer cells. Cells were treated with 5 μM NanoVP for 72 h. Flow cytometry was used to measure the intracellular fluorescence of rhodamine 123 (a, b) and calcein (c, d) in parental cells and rhodamine 123 (e, f) and calcein (g, h) in drug‐resistant cells. Data presented as representative histograms with the untreated control as the black trace and the NanoVP‐treated group as the magenta trace and bar graphs presented as mean ± SD values (*n* = 3), one‐tailed *t*‐test, Mann–Whitney test, *p* values are presented on plots; black is the control and magenta is the experimental group.

Since ATP levels were significantly decreased and VP uptake was comparable in drug‐resistant cells compared to their parental cell lines, we next sought to determine whether prolonged NanoVP exposure (5 μM NanoVP at 72 h) increased the intracellular accumulation of P‐gp substrates in drug‐resistant cells. We used flow cytometry to measure the levels of intracellular accumulation of fluorescent P‐gp substrates rhodamine 123 and calcein‐acetoxymethyl (AM) (measuring the fluorescence of calcein after cleavage of the AM ester group by intracellular esterases). After a 72‐h treatment with 5 μM NanoVP, we found that there were no significant differences in the accumulation of rhodamine 123 in MDA‐MB‐231 cells (Figure 5a), resulting in a fold change of 1.06 (Figure 5b). We found a decrease in calcein accumulation in MDA‐MB‐231 cells (Figure 5c), resulting in a fold change of 0.97, which was a statistically significant difference (Figure 5d). This indicates calcein leakage from MDA‐MB‐231 cells. We observed an increase in rhodamine 123 accumulation in VBL‐MDA‐MB‐231 cells (Figure 5e), resulting in a 1.63‐fold increase in accumulation (Figure 5f). Calcein accumulation was significantly increased in VBL‐MDA‐MB‐231 cells (Figure 5g) post‐treatment, resulting in a 5.1‐fold increase in accumulation (Figure 5h).

**FIGURE 6:**
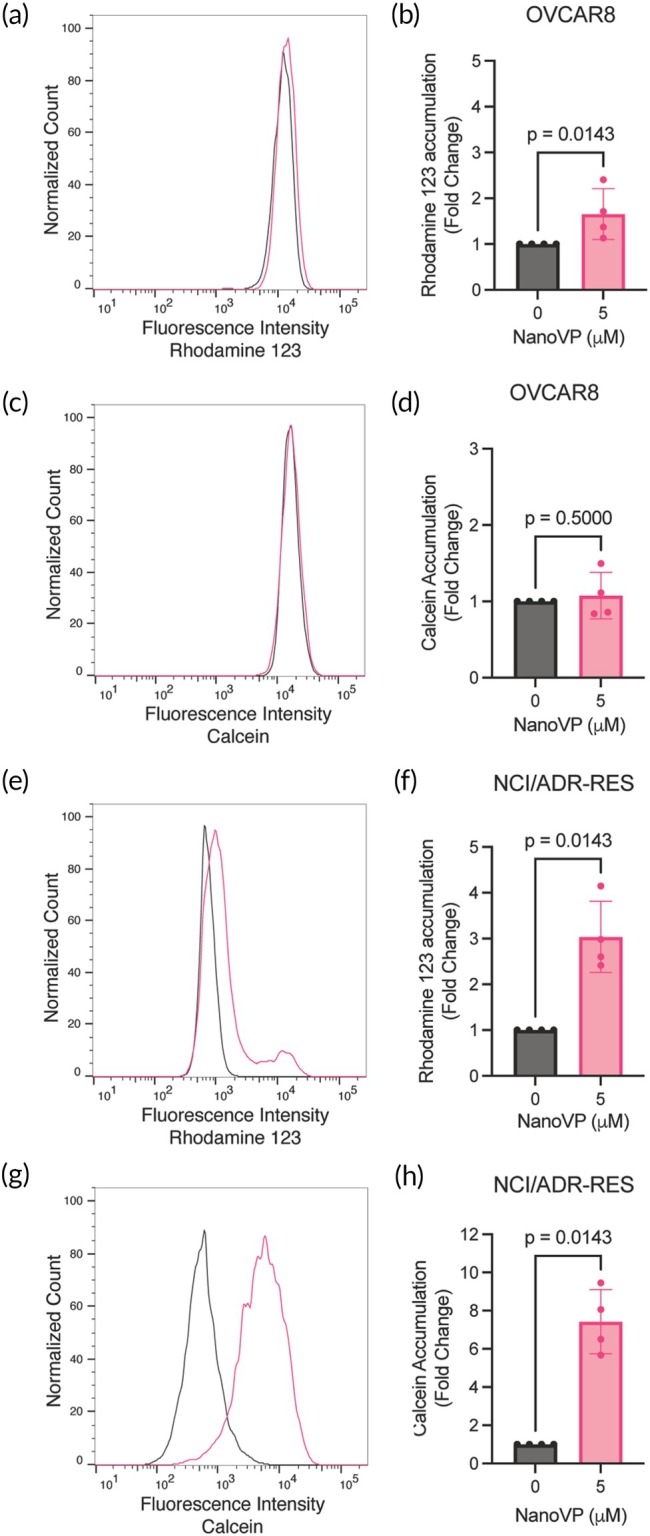
Nanoaggregates of verteporfin (NanoVP) treatment increases the accumulation of P‐glycoprotein substrates after 72 h in drug‐resistant ovarian cancer cells. Cells were treated with 5 μM NanoVP for 72 h. Flow cytometry was used to measure the intracellular fluorescence of rhodamine 123 (a, b) and calcein (c, d) in parental cells and rhodamine 123 (e, f) and calcein (g, h) in drug‐resistant cells. Data presented as representative histograms with the untreated control as the black trace and the NanoVP‐treated group as the magenta trace and bar graphs presented as mean ± SD values (*n* = 3), one‐tailed *t*‐test, Mann–Whitney test, *p* values are presented on plots; black is the control and magenta is the experimental group.

After a 72‐h treatment with 5 μM NanoVP, we observed an increase in rhodamine 123 accumulation in OVCAR8 cells (Figure 6a), resulting in a 1.7‐fold increase (Figure 6b). We did not observe a significant difference in calcein accumulation in OVCAR8 cells (Figure 6c), resulting in a 1.08‐fold increase (Figure 6d). We observed a significant increase in rhodamine 123 accumulation in NCI‐ADR/RES cells (Figure 6e), resulting in a 3.04‐fold increase in accumulation (Figure 6f). Calcein accumulation also significantly increased in NCI‐ADR/RES cells (Figure 6g) post‐treatment, resulting in a 7.43‐fold increase in accumulation (Figure 6h).

The fold changes in rhodamine 123 and calcein accumulation in both parental cell lines remained close to 1, indicating minimal overall changes. This outcome was expected, given the lack of P‐gp expression in these cells. In contrast, rhodamine 123 and calcein accumulation increased in both drug‐resistant TNBC and ovarian cancer cells, consistent with impaired P‐gp‐mediated substrate efflux and enhanced intracellular substrate retention as a result of ATP depletion at those conditions. Notably, while retention of both substrates was significantly improved, the fold change in rhodamine 123 was lower (1.5 to 3‐fold higher compared to control) than that of calcein (five‐ to seven‐fold higher compared to control) between the drug‐resistant cell lines.

These differences are consistent with known differences in probe behavior following cellular uptake. Calcein‐AM is the transported species, directly effluxed from the membrane, and upon intracellular de‐esterification to calcein, it becomes membrane‐impermeable, allowing intracellular signal to accumulate even with partial impairment of efflux.^36^ In contrast, rhodamine 123 remains a P‐gp substrate following cellular uptake and its accumulation in mitochondria also partially depends on mitochondrial membrane potential (ΔΨm).^37^ To independently assess changes in ΔΨm, we measured tetramethylrhodamine ethyl ester (TMRE) fluorescence following a 72‐h treatment of 5 μM NanoVP and found that ΔΨm was significantly depolarized in drug‐resistant cells (Figure S1). TMRE was used as an independent and highly ΔΨm‐sensitive probe to directly measure mitochondrial depolarization, as it exhibits strong ΔΨm‐dependent mitochondrial accumulation with minimal ΔΨm‐independent binding, making it a sensitive reporter of changes in mitochondrial membrane potential compared to rhodamine derivatives.^37^, ^38^ In contrast, rhodamine 123 displays mixed binding behavior with both ΔΨm‐dependent mitochondrial accumulation and ΔΨm‐independent membrane association, allowing modest intracellular fluorescence to persist even under depolarized conditions. Accordingly, the attenuated yet detectable increase in rhodamine 123 accumulation—relative to calcein—likely reflects the combined influence of reduced P‐gp‐mediated efflux and diminished mitochondrial sequestration.^37^, ^38^ For this reason, calcein‐AM and rhodamine 123 were used as complementary probes with distinct intracellular behaviors to provide a more robust assessment of P‐gp functional changes under prolonged NanoVP exposure.

### 2.5 Prolonged treatment for 72 h with NanoVP affects UIC2 binding to P‐gp

Liang et al. demonstrated that VP interacts with P‐gp at the substrate‐binding pocket in the transmembrane domain and light activation of VP resulted in aggregation of P‐gp in insect membrane vesicles.^9^ In our previous work, we used MRK16, a P‐gp‐specific antibody, to measure changes in P‐gp surface expression levels in mammalian drug‐sensitive and drug‐resistant cancer cells and found that light activation of VP did not alter MRK16 binding, indicating no observable changes in P‐gp expression levels.^10^ In the present study, we wanted to understand how a 72‐h treatment with 5 μM NanoVP without light activation alters the binding of UIC2, a P‐gp conformation‐specific monoclonal antibody. This assay assesses how the longer incubation with a higher concentration of NanoVP impacts P‐gp expression levels at the cell surface and whether sustained NanoVP exposure impacts P‐gp conformation in a way that directly inhibits drug efflux.^9^ To optimize UIC2 binding, we pre‐treated cells with cyclosporine A (CsA), which stabilizes P‐gp in an inward‐facing conformation.^39^, ^40^ The conformation sensitivity of UIC2 is shown by increased binding after the addition of CsA (Figure S2). We therefore evaluated whether prolonged NanoVP exposure alters this CsA‐dependent change in UIC2 binding.

**FIGURE 7:**
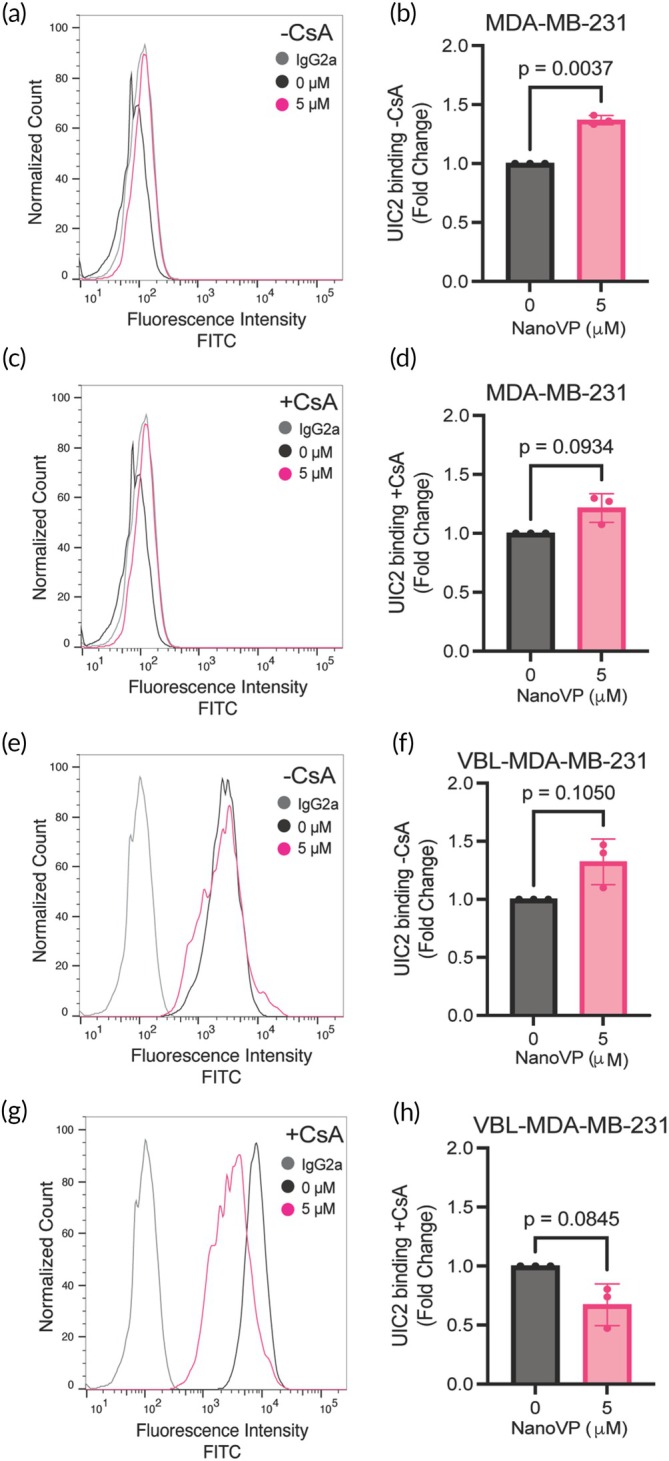
Nanoaggregates of verteporfin (NanoVP) treatment alters UIC2 binding to P‐glycoprotein in drug‐resistant triple‐negative breast cancer cells. Cells were treated with 5 μM NanoVP for 72 h. Flow cytometry was used to measure the UIC2 binding without (a, b) and with (c, d) cyclosporine A (CsA) in parental cells, and without (e, f) and with (g, h) in drug‐resistant cells. Data presented as representative histograms with the IgG2a negative control as the gray trace, the untreated control as the black trace, and the NanoVP‐treated group as the magenta trace and bar graphs presented as mean ± SD values (*n* = 3), one‐tailed *t*‐test, Mann–Whitney test, *p* values are presented on plots; black is the control and magenta is the experimental group.

**FIGURE 8:**
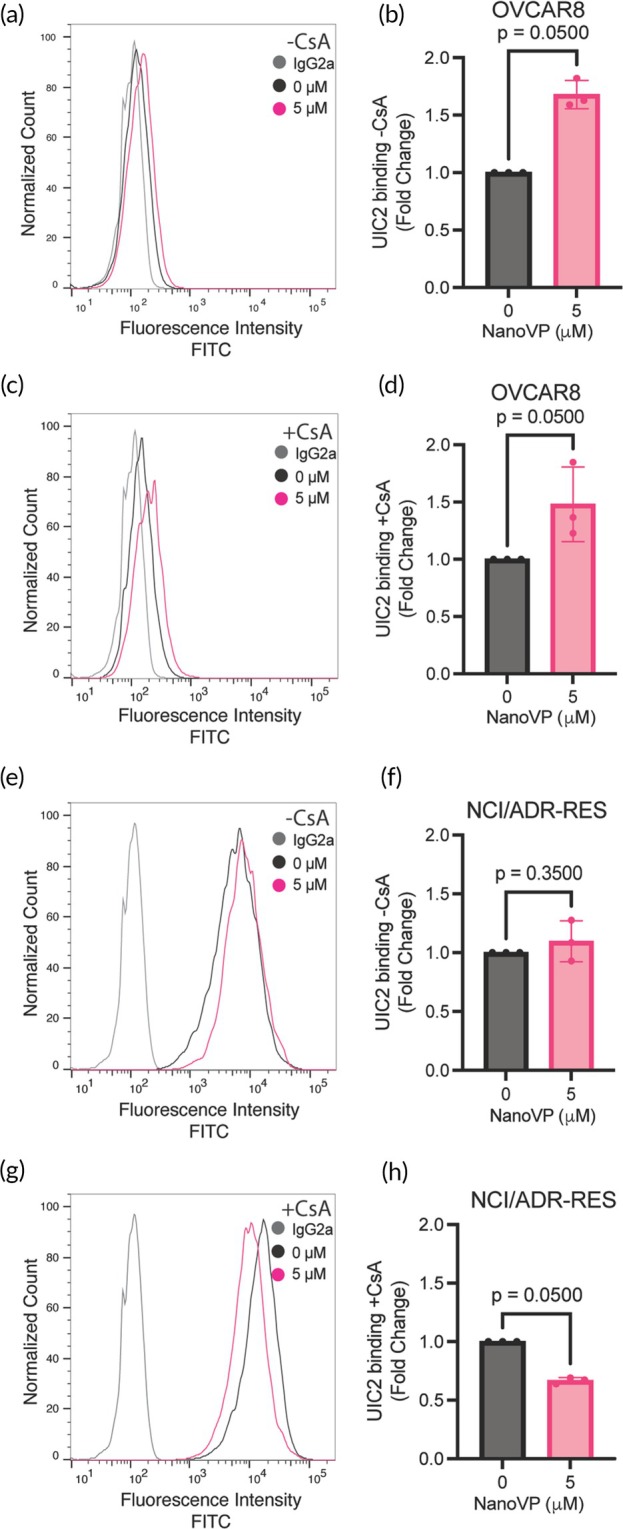
Nanoaggregates of verteporfin (NanoVP) treatment alters UIC2 binding to P‐glycoprotein in drug‐resistant ovarian cancer cells. Cells were treated with 5 μM NanoVP for 72 h. Flow cytometry was used to measure the UIC2 binding without (a, b) and with (c, d) cyclosporine A (CsA) in parental cells, and without (e, f) and with (g, h) in drug‐resistant cells. Data presented as representative histograms with the IgG2a negative control as the gray trace, the untreated control as the black trace and the NanoVP‐treated group as the magenta trace and bar graphs presented as mean ± SD values (*n* = 3), one‐tailed *t*‐test, Mann–Whitney test, *p* values are presented on plots; black is the control and magenta is the experimental group.

Following treatment with 5 μM NanoVP without CsA pre‐treatment, the UIC2 binding in MDA‐MB‐231 cells increased modestly (Figure 7a), resulting in a 1.37‐fold increase (Figure 7b). After pre‐treatment with CsA, UIC2 binding in MDA‐MB‐231 cells remained similar, with a 1.2‐fold increase in binding in the presence of NanoVP (Figure 7c,d). Without CsA pre‐treatment, the UIC2 binding to P‐gp on VBL‐MDA‐MB‐231 cells increased by 1.32‐fold in the presence of NanoVP (Figure 7e,f). In contrast, after pre‐treatment with CsA, UIC2 binding to P‐gp on VBL‐MDA‐MB‐231 cells decreased (Figure 7g), resulting in a fold change of 0.67 in the presence of NanoVP (Figure 7h). Without CsA pre‐treatment, UIC2 binding to OVCAR8 cells increased by 1.68‐fold (Figure 8a,b) and after pre‐treatment with CsA, UIC2 binding to OVCAR8 cells increased by 1.48‐fold (Figure 8c,d), both in the presence of NanoVP. Without CsA pre‐treatment, we did not observe changes in UIC2 binding to P‐gp on NCI‐ADR/RES cells (Figure 8e), resulting in a 1.09‐fold increase in binding (Figure 8f). After pre‐treatment with CsA, UIC2 binding to P‐gp on NCI‐ADR/RES cells decreased (Figure 8g), resulting in a fold change of 0.67 in the presence of NanoVP (Figure 8h).

As expected, low UIC2 binding in both parental cell lines was comparable to low binding of the mouse immunoglobulin G subclass 2a (IgG2a) isotype control, consistent with minimal P‐gp expression and fold changes of approximately 1 both in the absence and presence of CsA. In both drug‐resistant cell lines, we observed an increase in UIC2 binding in the absence of CsA pre‐treatment and a decrease in UIC2 binding in the presence of CsA pre‐treatment. One interpretation is that sustained exposure to 5 μM NanoVP alters P‐gp conformation and/or binding site occupancy in a manner that overlaps with or interferes with CsA‐stabilized conformational states. Since VP is known to occupy the substrate‐binding pocket in the transmembrane domain (the same site where CsA binds), NanoVP binding could prevent CsA from further stabilizing P‐gp in the inward‐facing conformation associated with maximal UIC2 binding.^9^, ^41^, ^42^ Another possible explanation for the decreased UIC2 binding after CsA treatment is that NanoVP inhibits P‐gp at the transcriptional or translational level, leading to reduced overall P‐gp expression. Further studies are needed to determine whether prolonged exposure to 5 μM NanoVP decreases total P‐gp protein levels. Overall, when considered alongside the observed ATP depletion and reduced efflux activity at 72 h, changes in UIC2 binding are consistent with a model in which sustained NanoVP exposure impairs P‐gp function through a combination of ATP‐dependent effects and transporter‐level modulation. Further studies are required to distinguish if the transporter‐level effects are specifically due to conformational effects, substrate‐binding competition, and changes in P‐gp expression.

### 2.6 Combination treatment with NanoVP improves chemotherapy response in drug‐resistant cells

The substrate accumulation assay demonstrated partial inhibition of P‐gp following treatment with 5 μM NanoVP for 72 h, as indicated by increased accumulation of rhodamine 123 and calcein in drug‐resistant cells compared to their parental counterparts. To determine whether this partial inhibition was sufficient to enhance chemotherapy sensitization in drug‐resistant cells, we evaluated the IC_50_ values of both the drug‐selection agents used to induce acquired resistance (vinblastine for VBL‐MDA‐MB‐231 cells and doxorubicin for NCI‐ADR/RES cells) and irinotecan, a chemotherapeutic agent with a distinct mechanism of action that is also a P‐gp substrate. We measured how the observed changes in P‐gp function and UIC2 binding affected the sensitization of cells to chemotherapy by co‐treating cells with 5 μM NanoVP and each chemotherapeutic agent for 72 h. NanoVP was present continuously throughout the 72‐h chemosensitivity assays, allowing metabolic perturbation and efflux impairment to develop during chemotherapy exposure rather than requiring a discrete pre‐treatment phase. Thus, vinblastine and doxorubicin were chosen because they are the reagents that originally established the resistant phenotypes in TNBC and ovarian cancer cell pairs, respectively, while irinotecan was included to test whether NanoVP‐mediated sensitization extended to an unrelated P‐gp substrate.

**FIGURE 9:**
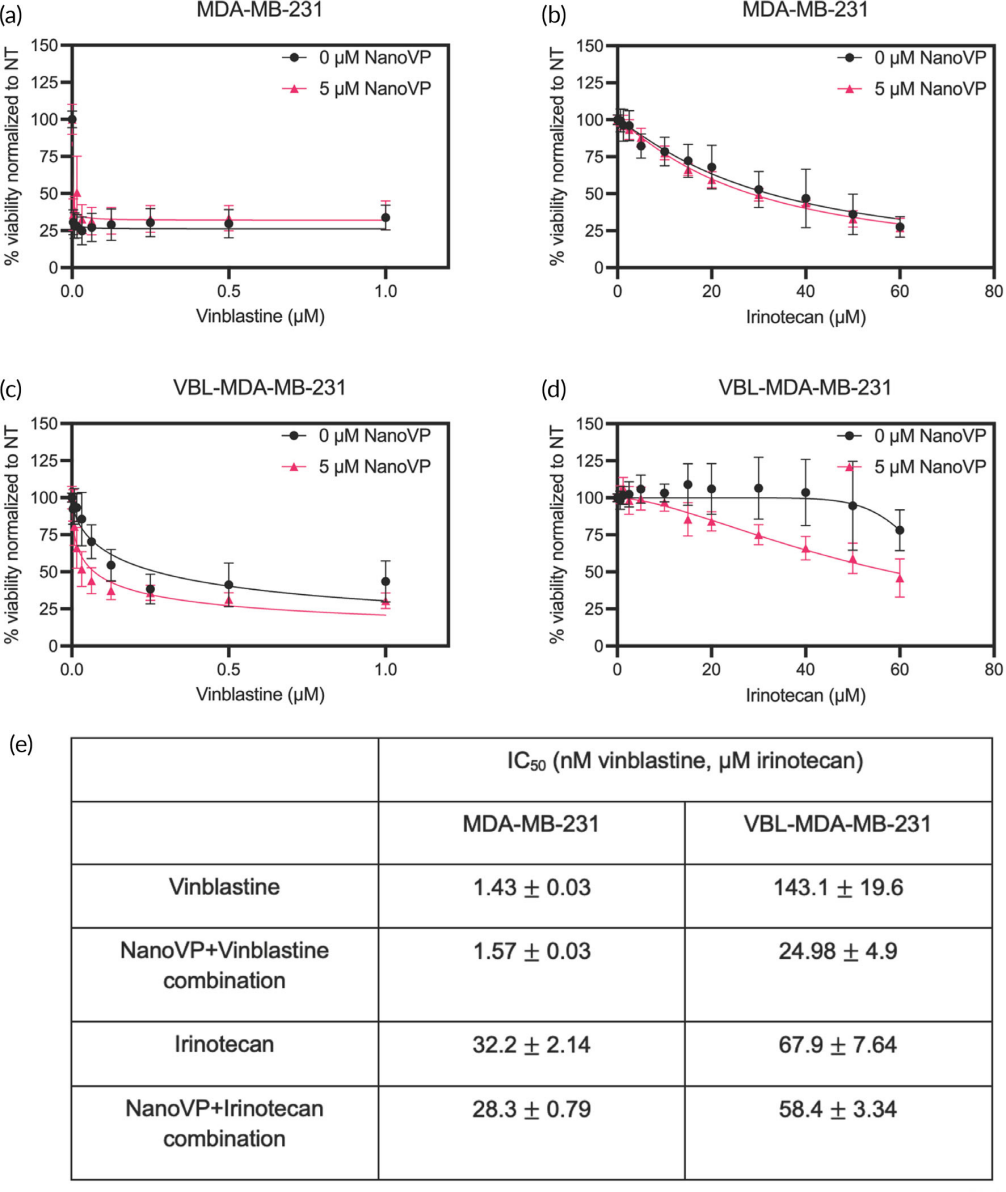
Combination treatment with 5 μM nanoaggregates of verteporfin (NanoVP) enhances chemotherapy efficacy in drug‐resistant triple‐negative breast cancer cells. Cells were treated with a single chemotherapy treatment (either vinblastine or irinotecan) or the combination of 5 μM NanoVP with chemotherapy for 72 h. CellTiter‐Fluor was used to measure the cell viability after vinblastine treatment in MDA‐MB‐231 cells (a), irinotecan treatment in MDA‐MB‐231 cells (b), vinblastine treatment in VBL‐MDA‐MB‐231 cells (c), and irinotecan treatment in VBL‐MDA‐MB‐231 cells (d). IC_50_ values for chemotherapy treatment for single and the combination treatment with 5 μM NanoVP after 72 h in MDA‐MB‐231 cells and VBL‐MDA‐MB‐231 cells are summarized (e). Data presented as mean ± SD values (*n* = 3), where black is the single chemotherapy treatment and magenta is the combination treatment.

In MDA‐MB‐231 cells, the IC_50_ of the vinblastine alone treatment was 1.43 ± 0.03 nM and after the combination treatment with 5 μM NanoVP, the IC_50_ of vinblastine was 1.57 ± 0.03 nM (Figure 9a). The IC_50_ of the irinotecan alone treatment was 32.23 ± 2.140 μM and after the combination treatment, the IC_50_ of irinotecan was 28.32 ± 0.794 μM (Figure 9b). In VBL‐MDA‐MB‐231 cells, the IC_50_ of the vinblastine alone treatment was 143.1 ± 19.6 nM, and the IC_50_ of vinblastine after the combination treatment with 5 μM NanoVP was 24.98 ± 4.9 nM (Figure 9c). The IC_50_ of the irinotecan alone treatment was 67.93 ± 7.637 μM and after the combination treatment, the IC_50_ of irinotecan was 58.43 ± 3.344 μM (Figure 9d). The values are summarized in Figure 9e.

**FIGURE 10:**
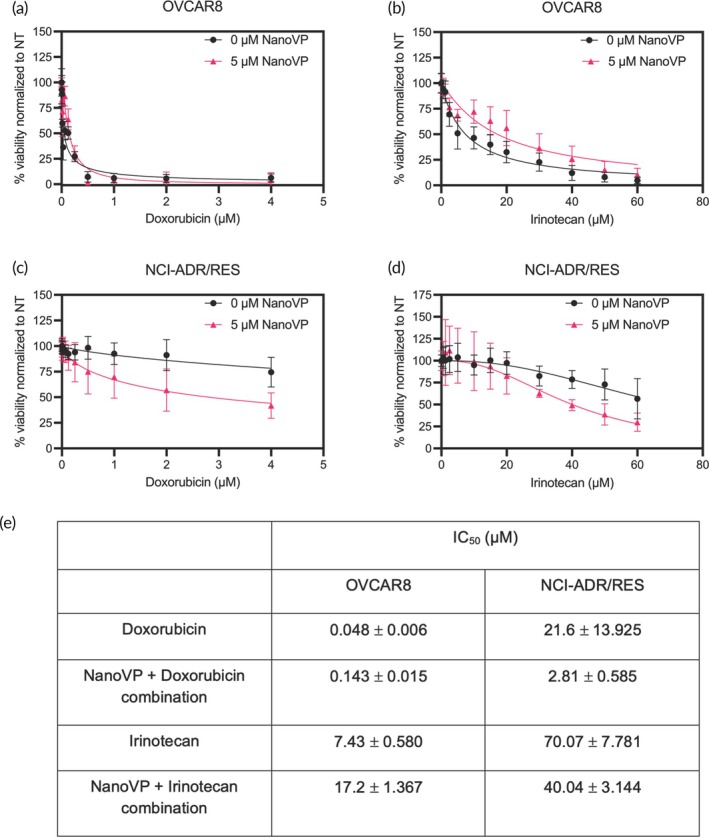
Combination treatment with 5 μM nanoaggregates of verteporfin (NanoVP) enhances chemotherapy efficacy in drug‐resistant ovarian cancer cells. Cells were treated with a single chemotherapy treatment (either doxorubicin or irinotecan) or the combination of 5 μM NanoVP with chemotherapy for 72 h. CellTiter‐Fluor was used to measure the cell viability after doxorubicin treatment in OVCAR8 cells (a), irinotecan treatment in OVCAR8 cells (b), doxorubicin treatment in NCI‐ADR/RES cells (c), and irinotecan treatment in NCI‐ADR/RES cells (d). IC_50_ values for chemotherapy treatment for single and the combination treatment with 5 μM NanoVP after 72 h in OVCAR8 cells and NCI‐ADR/RES cells are summarized (e). Data presented as mean ± SD values (*n* = 3), where black is the single chemotherapy treatment and magenta is the combination treatment.

In OVCAR8 cells, the IC_50_ of the single doxorubicin treatment was 0.048 ± 0.006 μM and after the combination treatment with 5 μM NanoVP the IC_50_ of doxorubicin was 0.143 ± 0.015 μM (Figure 10a). The IC_50_ of the single irinotecan treatment was 7.425 ± 0.580 μM and after the combination treatment, the IC_50_ of irinotecan was 17.2 ± 1.367 μM (Figure 10b). In NCI‐ADR/RES cells, the IC_50_ of the single doxorubicin treatment was predicted to be 21.6 ± 13.925 μM, and the IC_50_ of doxorubicin after the combination treatment with 5 μM NanoVP was 2.81 ± 0.585 μM (Figure 10c). The IC_50_ of the single irinotecan treatment was predicted to be 70.07 ± 7.781 μM and after the combination treatment, the IC_50_ of irinotecan was 40.04 ± 3.144 μM (Figure 10d). The values are summarized in Figure 10e.

Vinblastine, a *vinca* alkaloid, disrupts the mitotic spindle and arrests cell division at metaphase.^43^ Doxorubicin, an anthracycline, exerts its cytotoxic effects through two primary mechanisms: intercalation into DNA to disrupt topoisomerase II‐mediated DNA repair and the generation of free radicals that damage cellular membranes, DNA, and proteins.^44^ Irinotecan, on the other hand, inhibits topoisomerase I‐mediated DNA repair.^45^ Across both MDR models, co‐treatment with NanoVP reduced IC_50_ values for the selection drug and irinotecan in the drug‐resistant sublines, consistent with improved response to P‐gp substrate chemotherapeutics under conditions where NanoVP exposure impairs efflux function. In contrast, in the parental cell lines, NanoVP co‐treatment increased IC_50_ values for some drugs, suggesting that metabolic perturbation by NanoVP may influence drug response independently of P‐gp‐mediated efflux in cells that do not rely on active efflux for drug resistance. Importantly, this differential response underscores the context‐dependent therapeutic intent of NanoVP. The goal of NanoVP administration is not to enhance chemosensitivity in non‐resistant cells, but rather to selectively target metabolic and efflux vulnerabilities in multidrug‐resistant, P‐gp‐overexpressing tumors. In parental cell lines lacking P‐gp overexpression, NanoVP‐induced metabolic perturbation may influence chemotherapy response through transporter‐independent mechanisms, underscoring that the chemosensitizing effect of NanoVP is specific to MDR contexts rather than universally applicable. Within this context, NanoVP‐mediated ATP depletion and efflux impairment provide a rational strategy for resensitizing resistant cancer cells to standard chemotherapeutics. Future studies will be needed to optimize treatment scheduling (e.g., metabolic preconditioning versus concurrent administration) and to further disentangle transporter‐dependent and transporter‐independent contributions to NanoVP‐mediated chemosensitization.

### 2.7 Limitations and future directions

Despite its strengths, this study has several limitations that should be acknowledged. First, although prior work has implicated YAP‐TEAD signaling in light‐independent VP activity, Hippo pathway signaling was not directly investigated here. Therefore, the relationship between NanoVP‐induced metabolic perturbation and YAP‐TEAD modulation remains unresolved. Next, Seahorse‐based metabolic assays provide functional readouts of cellular respiration and ATP production but do not allow definitive assignment of direct molecular targets within the ETC, and additional biochemical assays would be required to resolve specific site(s) of action. Additionally, all experiments were performed in vitro using established cancer cell lines, and in vivo pharmacokinetics, biodistribution, and toxicity of NanoVP were not evaluated. Finally, chemotherapy sensitization was evaluated using a single treatment schedule, and alternative dosing paradigms (e.g., NanoVP pre‐treatment followed by chemotherapy) were not explored. Despite these limitations, the present study provides mechanistic insight into light‐independent NanoVP‐mediated metabolic stress and functional P‐gp inhibition in drug‐resistant cancer cells, establishing a foundation for future translational investigation.

Future studies should therefore investigate optimized VP delivery strategies in vivo, including intratumoral and intraperitoneal administration, to maintain therapeutically relevant drug concentrations at tumor sites. Given the high rates of chemoresistance observed clinically—affecting approximately 30%–50% of patients with TNBC and 50%–70% of patients with ovarian cancer^46^, ^47^—further preclinical studies are warranted to evaluate NanoVP as an adjuvant strategy for resensitizing drug‐resistant tumors to standard chemotherapy regimens.

## 3 CONCLUSIONS

In this study, we investigated the light‐independent effects of self‐assembled NanoVP on P‐gp inhibition. Our findings suggest that sustained exposure to NanoVP is associated with impaired P‐gp efflux activity, coinciding with pronounced ATP depletion and altered transport behavior at the plasma membrane. The increased accumulation of P‐gp substrates and enhanced sensitization of drug‐resistant cells to chemotherapy highlight VP's therapeutic potential beyond its traditional role as a photosensitizer. Collectively, these findings support the concept that repurposing VP in a light‐independent context may offer a complimentary strategy to existing approaches for overcoming MDR.

## 4 MATERIALS AND METHODS

### 4.1 Materials

The fluorescent compounds calcein‐AM and rhodamine 123 were obtained from Invitrogen (Carlsbad, CA) and Sigma‐Aldrich (St. Louis, MO), respectively. The photosensitizer VP was purchased from U.S. Pharmacopeia (Rockville, MD) and dissolved in dimethyl sulfoxide (DMSO), which was sourced from Sigma‐Aldrich (St. Louis, MO). All other chemicals and reagents were acquired from Thermo Fisher Scientific (Waltham, MA) or Sigma‐Aldrich (St. Louis, MO).

### 4.2 Preparation of NanoVP

NanoVP (condition 4—smallest nanoparticles with the highest VP concentration) was prepared using the protocol from Quinlan et al.^18^ Briefly, VP was first dissolved in DMSO, then added dropwise (10 μL) from a height of 15 cm into rapidly stirred ultrapure water (1500 revolutions per minute (RPM)) to facilitate the formation of self‐assembled J‐type nanoaggregates. The resulting nanoparticles were then dialyzed against 1× PBS to remove excess solvent. Nanoparticle size, PDI, and zeta potential were measured using a NanoBrook Omni instrument (Brookhaven). UV–Vis spectroscopy was employed to determine VP concentration.^18^, ^19^

### 4.3 Cell culture

The human female TNBC cell line MDA‐MB‐231 (Research Resource Identifier (RRID): CVCL_0062) was obtained from the National Cancer Institute.^48^ The vinblastine‐resistant subline (VBL‐MDA‐MB‐231) was established by continuously culturing MDA‐MB‐231 cells in 100 ng/mL vinblastine sulfate salt (Sigma Aldrich).^49^ MDA‐MB‐231 cells were maintained in Dulbecco's Modified Eagle Medium (DMEM) (Gibco), and VBL‐MDA‐MB‐231 cells were cultured in Roswell Park Memorial Institute (RPMI) 1640 Medium (Gibco). Both media were supplemented with 10% (v/v) fetal bovine serum (FBS), 100 U/mL penicillin, and 100 μg/mL streptomycin. All cell lines were routinely tested and confirmed to be mycoplasma‐free (MycoAlert, Lonza) and were maintained at 37°C in a humidified incubator with 5% CO_2_.

The human female ovarian cancer cell line OVCAR8 (RRID: CVCL_1629) was obtained from the Division of Cancer Treatment and Diagnosis Tumor Repository, National Cancer Institute (Frederick, MD). The doxorubicin‐resistant subline (NCI‐ADR/RES (RRID: CVCL‐1452)) was generated by continuous culture of OVCAR8 cells in 100 ng/mL doxorubicin‐hydrochloride (Sigma Aldrich). Both cell lines were cultured in RPMI 1640 Medium (Gibco), supplemented with 10% (v/v) FBS, 100 U/mL penicillin, and 100 μg/mL streptomycin. All cell lines were routinely tested and confirmed to be mycoplasma‐free (MycoAlert, Lonza) and were maintained at 37°C in a humidified incubator with 5% CO_2_.

### 4.4 Seahorse assay

Two Agilent Seahorse assay kits were used in this work to measure metabolic changes after NanoVP treatment: the RealTime ATP assay kit and the MitoStress Test kit. Different cell densities were seeded for each time point and for each assay. Forty thousand cells for 24 h, 20,000 cells for 48 h and 10,000 cells for 72 h were respectively seeded into the Seahorse 96‐well microplate and were allowed to attach overnight in 5% CO_2_ at 37°C. The next day, the cells were treated with 0–40 μM NanoVP (for the MitoStress Test assay, 0–20 μM were screened for the RealTime ATP assay) for the respective time points and left to incubate in 5% CO_2_ at 37°C. The Seahorse utility plate was hydrated the night before the assay would be performed with Ultrapure water (Invitrogen) at 37°C in a 0% CO_2_ environment, and the XF calibrant was also incubated under the same conditions. On the day of the assay, the XF calibrant was added to the utility plate and incubated at 37°C in 0% CO_2_ until ready for inhibitor loading. Seahorse DMEM was supplemented with glutamate (2 mM), pyruvate (1 mM), and glucose (10 mM). After incubation, cells were washed twice with PBS containing calcium and magnesium, and fresh Seahorse DMEM was added to each well. The cells were then incubated for 1 h at 37°C in 0% CO_2_ for degassing. For the RealTime ATP assay, ETC inhibitors oligomycin (15 μM) and rotenone/antimycin A (5 μM), were prepared and loaded onto the utility plate. For the MitoStress test kit, ETC inhibitors, oligomycin (15 μM), carbonyl cyanide *p*‐(trifluoromethoxy) phenylhydrazone (FCCP) (20 μM), and rotenone/antimycin A (5 μM), were prepared and loaded onto the utility plate. After the 1‐h incubation, the degassing medium was replaced with fresh Seahorse DMEM in each well (180 μM). The Seahorse analyzer was calibrated using the utility plate with the ETC inhibitors injected, after which the cell microplate was loaded into the instrument for metabolic measurement. To normalize Seahorse output (OCR, ECAR, mitoATP, glycoATP, ATP‐production‐coupled respiration) measurements to cell number, cells were fixed immediately following the assay with 4% neutral buffered formalin, followed by nuclei staining with NucBlue (Thermo Fisher) for post‐assay nuclear counting. Whole‐well imaging was performed at 4× magnification using the BioTek Lionheart Imager, and nuclei were quantified using Gen5 software. All Seahorse parameters were normalized to post‐assay nuclei counts.

### 4.5 Cell viability assays

CellTiter‐Fluor from Promega (Madison, WI) was used to measure the cell viability after NanoVP treatment for 24–72 h. Different cell densities were seeded for each time point in a 96‐well microplate: 40,000 cells for 24 h, 20,000 cells for 48 h, and 10,000 cells for 72 h. Cells were allowed to attach overnight in a humidified incubator at 37°C with 5% CO₂. The following day, cells were treated with 0–40 μM NanoVP for their respective time points and incubated under the same conditions (37°C, 5% CO_2_). On the day of the assay, cells were washed twice with PBS containing ions and 100 μL of fresh media was added to each well. The CellTiter‐Fluor assay buffer was prepared by adding 1 μL of the glycyl‐phenylalanyl‐amino‐fluorocoumarin (GF‐AFC) substrate per 1 mL of assay buffer (e.g., 10 μL GF‐AFC substrate into 10 mL assay buffer). A 1:1 mixture of cell culture medium and the pre‐prepared assay buffer was added to each well, followed by incubation at 37°C for 2 h under constant shaking. The fluorescent product, generated upon cleavage by live‐cell proteases, was detected using a BioTek Synergy Neo2 plate reader with an excitation wavelength of 390 nm and an emission wavelength of 505 nm.

Cell viability was also measured after combination treatment with chemotherapeutic agents, doxorubicin and vinblastine. For all time points, 6000 cells were seeded into a 96‐well microplate. The next day, cells were treated with 5 μM NanoVP and 0–1 μM vinblastine, or 0–5 μM doxorubicin, or 0–60 μM or irinotecan for 72 h and incubated at 37°C and 5% CO_2_. On the day of the assay, the cells were washed twice and the CellTiter‐Fluor assay buffer was prepared as above. A 1:1 mixture of cell culture medium and the pre‐prepared assay buffer was added to each well and incubated at 37°C for 2 h under constant shaking. The fluorescent product was detected using a BioTek Synergy Neo2 plate reader with an excitation wavelength of 390 nm and an emission wavelength of 505 nm.

### 4.6 VP extraction assay

Intracellular VP accumulation was quantified by adapting a previously published method.^50^ Briefly, 100,000 cells were seeded into 35 mm dishes and allowed to incubate overnight for attachment at 5% CO_2_ in 37°C. The next day, cells were incubated with 2 and 5 μM of NanoVP formulation for 24–72 h at 5% CO_2_ in 37°C. Cells were washed twice with phosphate buffered saline without ions, then lysed using Radioimmunoprecipitation Assay (RIPA) buffer (Thermo Fisher Scientific, USA) for 1 h. Fluorescence signals of the VP formulations were measured using a BioTek Synergy Neo2 plate reader at VP's excitation/emission wavelengths of 435/689 nm. Protein concentration (mg/mL) was determined using the bicinchoninic acid (BCA) Protein Assay (Thermo Fisher Scientific). Intracellular VP concentrations were quantified using standard curves and normalized to total protein content as determined by the BCA assay. All experiments were performed in at least duplicate with duplicate measurements on three separate days (*n* = 3).

### 4.7 Flow cytometry

Flow cytometry was used to measure the intracellular fluorescence of rhodamine 123 and calcein (the fluorescent product after the AM ester group is cleaved from calcein‐AM by intracellular esterases).^10^ Briefly, 100,000 cells were seeded into 35 mm dishes and allowed to incubate overnight for attachment at 5% CO_2_ in 37°C. The next day, the cells were treated with 5 μM NanoVP for the respective time points (24, 48, or 72 h) and left to incubate in 5% CO_2_ at 37°C. Cells were washed once with PBS, then trypsinized and collected in IMDM supplemented with 5% (v/v) FBS (Gibco). Cells were counted and 300,000 cells were aliquoted into 5 mL round‐bottom polystyrene tubes.

To investigate P‐gp function, harvested cells were incubated with P‐gp substrates: rhodamine 123 (1.3 μM) for 45 min or calcein‐AM (0.5 μM) for 10 min at 37°C.^51^ After being washed with cold IMDM, cells were resuspended in PBS containing 1% bovine serum albumin (BSA). One microliter of 0.2 mg/mL of DAPI (Sigma Aldrich) in DI water was added directly to each tube as a dead cell exclusion dye (final concentration 0.001 μg/μL). Flow cytometry analysis was conducted using a FACSCelesta (BD Biosciences) to assess substrate retention.^52^ Flow cytometry data were analyzed using FlowJo software, and dead cells were gated out based on DAPI staining (Tree Star, Inc., Ashland, OR). Calcein‐AM and rhodamine 123 were selected as fluorescent probes to assess P‐gp efflux function because both substrates are well established and widely used in flow cytometry–based assays of P‐gp activity.^53^, ^54^ Calcein‐AM is actively transported from the membrane by P‐gp and, following intracellular de‐esterification to calcein, becomes membrane‐impermeable, enabling sensitive detection of changes in efflux capacity, including partial inhibition.^36^ Rhodamine 123 is likewise a canonical P‐gp substrate, although its intracellular accumulation in mitochondria is additionally influenced by mitochondrial membrane potential.^37^ These complementary properties allow assessment of P‐gp functional changes under conditions of prolonged treatment and metabolic perturbation while also highlighting probe‐specific limitations that are considered in data interpretation.

To assess P‐gp expression, the human P‐gp‐specific antibody UIC2 was used, as it is conformation sensitive and provides information on the altered conformation of P‐gp due to VP. To enhance UIC2 binding, CsA (20 μM, final concentration) was used to stabilize P‐gp in the outward‐open state. Harvested cells were pre‐treated with or without CsA for 5 min at 37°C, followed by direct addition of UIC2 to the tube for 30 min at 37°C. An IgG2a isotype control was used as a negative control, with cells incubated for 1 h at 37°C. After incubation, cells were washed with cold (Iscove's Modified Dulbecco's Medium) IMDM (Gibco) by centrifugation at 1500 rpm for 5 min at 4°C. They were then incubated with an anti‐mouse FITC‐conjugated secondary antibody for 30 min at 37°C, followed by another IMDM wash before resuspension in PBS containing 1% BSA. To exclude dead cells, 1 μL of 0.2 mg/mL DAPI (4', 6‐diamidino‐2‐phenylindole) (Sigma Aldrich) in DI water was added to each tube (final concentration: 0.001 μg/μL).^10^ Flow cytometry was performed on a FACSCelesta (BD Biosciences) to quantify P‐gp surface expression, and FlowJo software was used for analysis. Dead cells were gated out based on DAPI staining (Tree Star, Inc., Ashland, OR).

## AUTHOR CONTRIBUTIONS

**IIdrisa Rahman** designed and performed experiments, analyzed data, and wrote the original draft of the manuscript. **Idrisa Rahman**, **Anju Meda**, **Kaitlyn A. Moore**, and **Andaleeb Sajid** performed experiments and contributed to data collection and analysis. **Suresh V. Ambudkar** and **Huang Chiao Huang** secured funding and provided overall project direction. All authors reviewed, edited, and approved the final version of the manuscript.

## FUNDING INFORMATION

This work was supported by the NCI‐UMD Partnership for Integrative Cancer Research. Idrisa Rahman was supported by the NCI‐UMD Partnership for Integrative Cancer Research. Andaleeb Sajid and Suresh V. Ambudkar were supported by the Intramural Research Program of the National Institutes of Health, National Cancer Institute, Center for Cancer Research (ZIABC010030). Kaitlyn A. Moore was supported by the Clark Doctoral Fellowship. This work was also supported by the National Institutes of Health grants (R01CA260340, R01CA256710, and R01NS106229), University of Maryland Bioengineering Translational Initiative Grant, and UM Greenebaum Comprehensive Cancer Center (UMGCCC) Pilot Grant to Huang Chiao Huang.

## CONFLICT OF INTEREST STATEMENT

The authors declare that they have no known competing financial interests or personal relationships that could have appeared to influence the work reported in this paper.

## Supporting information


**Data S1.** Supporting Information.

## Data Availability

All the data supporting the findings of this study are available within the article and Supporting Information S1 from the corresponding author.
