# Supplementary material for: Self‐assembled verteporfin nanoparticles decrease drug efflux by P‐glycoprotein without light activation in drug‐resistant cancer cells
Source: Bioeng Transl Med. 2026 Mar 20;11(4):e70136. doi: 10.1002/btm2.70136 (PMC13327617; doi:10.1002/btm2.70136)
Supplement: Supplementary file 1 — Data S1. Supporting Information. [file BTM2-11-e70136-s001.docx]

**Supplementary information for:**

**Self-assembled verteporfin nanoparticles decrease drug efflux by P-glycoprotein without light activation in drug-resistant cancer cells**

Idrisa Rahman^1 ,2^ Anju Meda^1^, Kaitlyn A. Moore^1^, Andaleeb Sajid^2^, Suresh V. Ambudkar^2^, Huang-Chiao Huang^1,3^

^1^ Fischell Department of Bioengineering, University of Maryland, College Park, MD 20742, USA.

^2^ Laboratory of Cell Biology, Center for Cancer Research, National Cancer Institute, National Institutes of Health, Bethesda, MD 20892

^3^Marlene and Stewart Greenebaum Cancer Center, University of Maryland School of Medicine, Baltimore, MD 21201, USA

*Corresponding authors. Emails: [ambudkar@mail.nih.gov](mailto:ambudkar@mail.nih.gov), [hchuang@umd.edu](mailto:hchuang@umd.edu)

Addresses:

Laboratory of Cell Biology, Center for Cancer Research, NCI, NIH, 37 Convent Dr., Room 2120, Bethesda MD, 20892-4256

University of Maryland, A. James Clark Hall, 8278 Paint Branch Dr., College Park, MD, 20742

| **Supplementary Table 1.** | Physicochemical characterization of NanoVP |
| --- | --- |
| **Supplementary Figure 1.** | 72-hour treatment with 5 µM NanoVP decreases mitochondrial membrane potential in parental and drug-resistant cells. |
| **Supplementary Figure 2.** | Cyclosporine A (CsA) increases UIC2 binding to P-gp in drug-resistant cells. |

| Parameter | Mean ± SD | n | Method |
| --- | --- | --- | --- |
| Hydrodynamic diameter (nm) | 146 ± 26 | ≥3 | DLS |
| Polydispersity index (PDI) | 0.17 ± 0.05 | ≥3 | DLS |
| Zeta potential (mV) | -35.2 ± 11.1 | 2 | Electrophoretic light scattering |

**Supplementary Table 1. Physicochemical characterization of NanoVP.** Hydrodynamic diameter, polydispersity index (PDI), and zeta potential of self-assembled verteporfin nanoparticles (NanoVP) were measured by dynamic light scattering (DLS) and electrophoretic light scattering. Values are reported as mean ± SD from independent measurements.


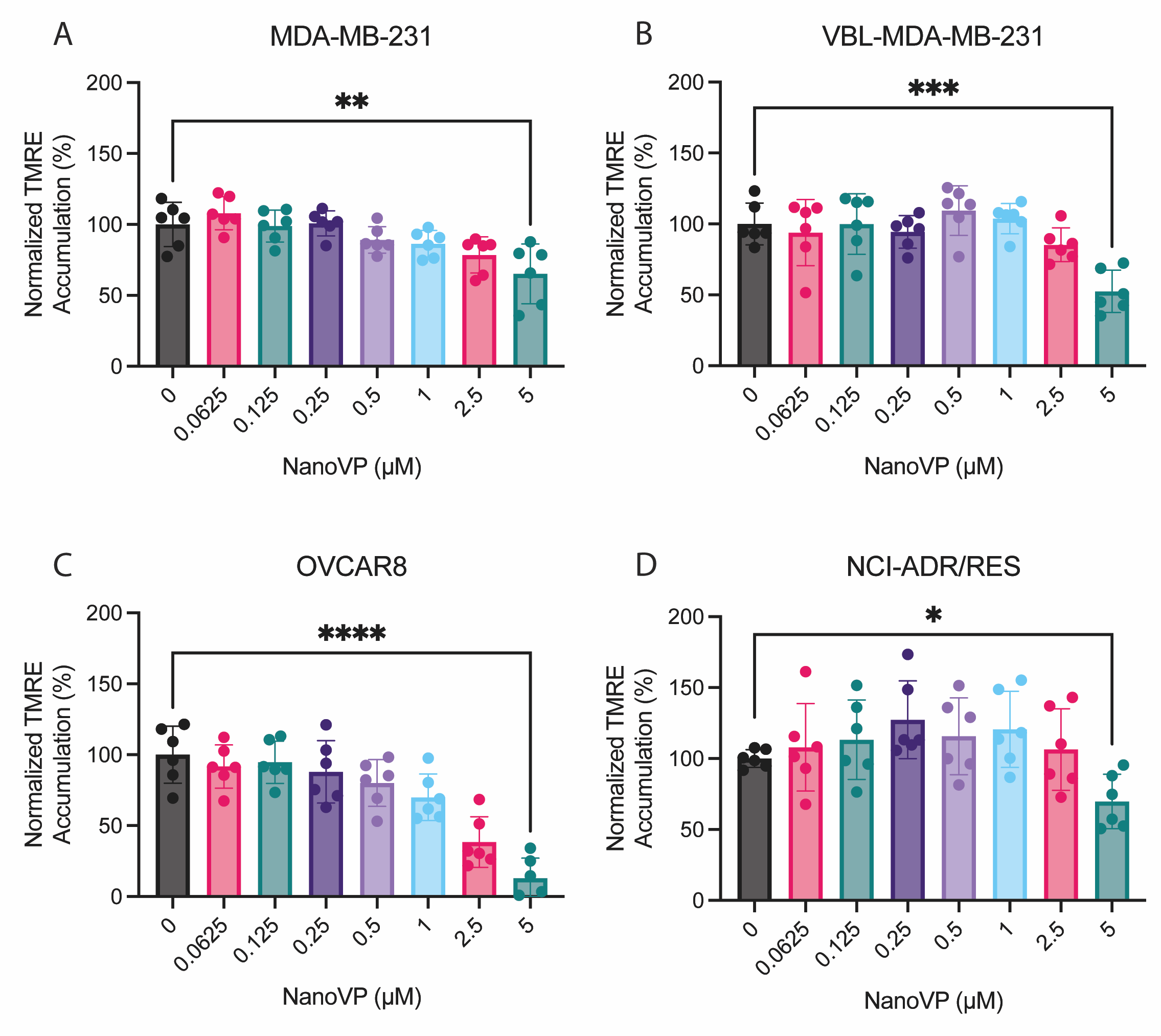


**Supplementary Figure 1. 72-hour treatment with 5 µM NanoVP decreases mitochondrial membrane potential in parental and drug-resistant cells.** A TMRE assay was used to measure the changes to mitochondrial membrane potential after cells were treated with 5 µM NanoVP for 72 hours in MDA-MB-231 cells (A), VBL-MDA-MB-231 cells (B), OVCAR8 cells (C) and NCI-ADR/RES cells (D). Data presented as mean ± SD values (n=2 in triplicate), *P≤ 0.05, **P ≤ 0.01, ***P≤ 0.001, ****P≤ 0.0001, two-tailed t-test.


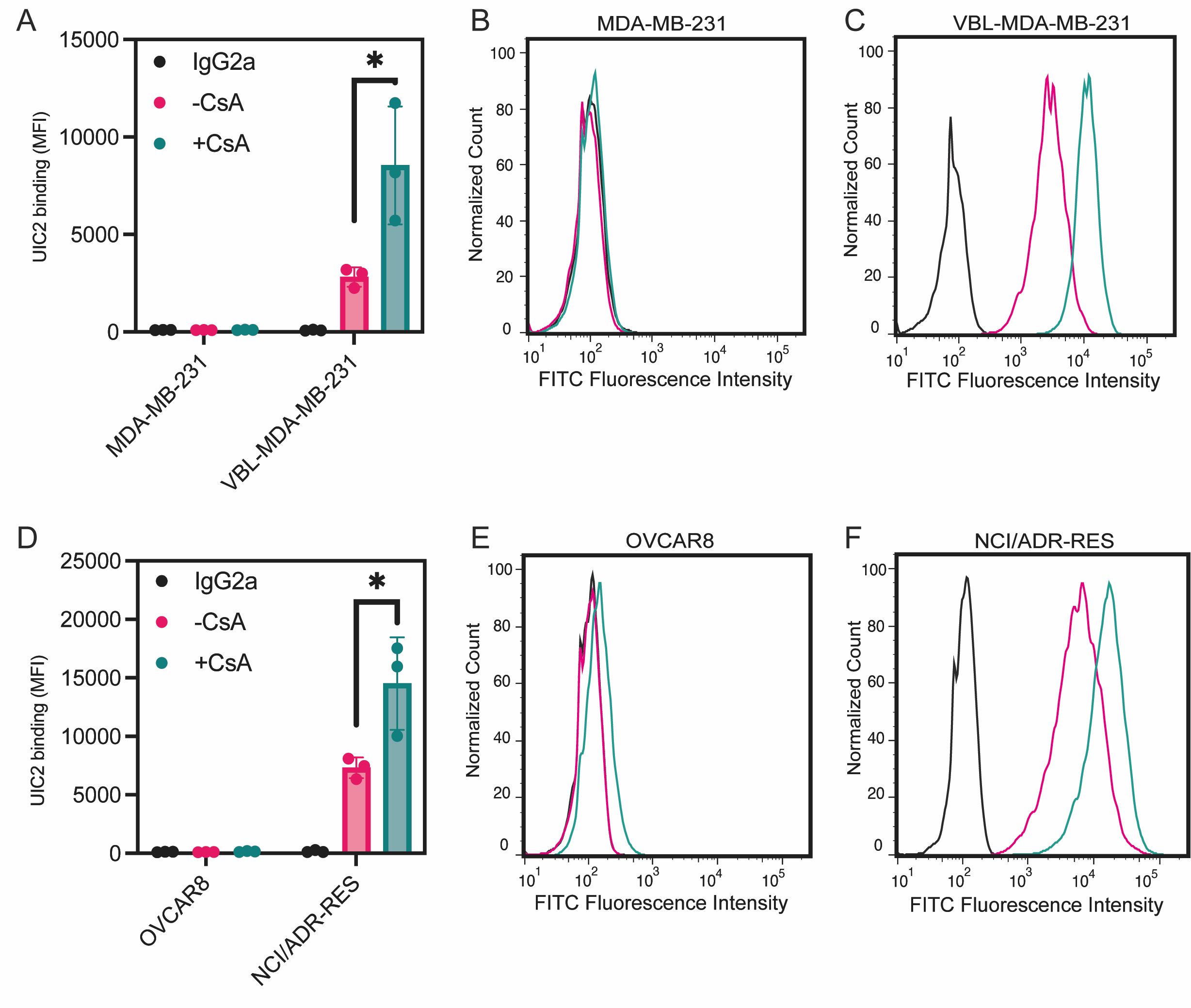


**Supplementary Figure 2. Cyclosporine A increases UIC2 binding to P-gp in drug-resistant cells.** Flow cytometry was used to measure the UIC2 binding without and with 20 µM cyclosporine A (CsA) compared to IgG2A binding in both cell lines in the TNBC cell pair (A) and ovarian cancer cell pair (D). Histograms show the differences in UIC2 binding with and without CsA compared to IgG2a in MDA-MB-231 cells (B) and OVCAR8 cells (E). Histograms show the differences in UIC2 binding with and without CsA compared to IgG2a in VBL-MDA-MB-231 cells (C) and NCI/ADR-RES cells (F). Data presented as bar graphs showing mean ± SD values (n=3), one-tailed t-test, Mann-Whitney test, *P≤ 0.05. Representative histograms show the IgG2a negative control as the black trace, the -CsA as the magenta trace and the +CsA as the teal trace.
